# Autologous full-thickness retinal transplant for refractory large macular holes

**DOI:** 10.1186/s40942-020-00266-5

**Published:** 2020-11-23

**Authors:** Sergio Rojas-Juárez, Javier Cisneros-Cortés, Abel Ramirez-Estudillo, Raul Velez-Montoya

**Affiliations:** 1 Retina Department, Hospital de Nuestra Señora de La Luz, 06030 Mexico City, Mexico; 2 Retina Department, Asociación para Evitar le Ceguera en México IAP, Vicente García Torres #46. Col: San Lucas Coyoacán, 04030 Mexico City, Mexico

**Keywords:** Macular hole, Autologous retinal transplant, Large macular hole, Refractory macular hole, Retina free patch

## Abstract

**Background:**

Despite the constant refinement of techniques and surgical aids, extremely large and refractory macular holes continue to have poor surgical outcomes with the current standard of care. The objective of the present study is to assess the anatomical and functional outcomes, as well as the structural change through time, of the optical coherence tomography of patients with refractory macular holes treated with a full-thickness autologous retinal transplant.

**Methods:**

Prospective, case series. We include patients with a clinical diagnosis of refractory macular holes with a minimum diameter of at least 500 µm. All the patients had a comprehensive ophthalmological examination, which included a best-corrected visual acuity assessment, fundus examination, and optical coherence analysis. All the patients underwent a 23-gauge pars plana vitrectomy with a full-thickness retinal transplant and silicone oil tamponade (5000 cs<). Follow-up was done at 1, 3, 6, and 12 months. Statistical analysis was done with a test for repeated measurements and Bonferroni correction, with an alpha value of 0.05 for statistical significance and a Mann-Whitney U test for nonparametric continuous variables.

**Results:**

We enrolled 13 eyes from 13 patients (mean age: 67.15 years) with refractory macular holes, with a mean base diameter of 1615.38 ± 689.19 µm and a minimum diameter of 964.08 ± 709.77 µm. The closure rate after 12 months of follow-up was 76.92%. Six patients with a closed macular hole at the end of the follow-up had complete recovery of the myoid/ellipsoid layer. The remaining showed a 44.9% reduction of the initial gap. Most patients formed a pseudofovea and normalization of the internal retinal layers. Despite a positive trend toward visual recovery (*p* = 0.034), after the correction of the alpha value, the change lost its statistical significance. During follow-up, one patient developed mild proliferative vitreoretinopathy and epiretinal membrane without anatomical or functional consequences.

**Conclusions:**

An autologous full-thickness retinal transplant may improve the anatomical and structural outcome of patients with refractory macular holes. The full safety profile of this new technique is still unknown. More studies are needed in order to assess functional changes through time.

## Background

Through the last decade, the surgical prognosis of an idiopathic macular hole (MH) has improved significantly, mainly because of the constant refinement of surgical techniques as well as the development of new and specialized surgical instruments and dyes [1–3].

Such improvements have encouraged retinal surgeons to try expanding surgical indications to MH with clinical characteristics associated with poorer prognosis (MH associated with high myopia, or retinal detachment, inflammatory diseases, and extremely large macular holes [2, 4, 5].

The autologous full-thickness retinal transplant (ART) is a novel surgical technique proposed by Grewal and Mahmoud that aims to treat patients when other surgical options are not feasible or are known to have a poorer prognosis [large macular holes with multiple previous retinal detachments, and primary closure failure (refractory) with extensive internal limiting membrane (ILM) peeling] [6, 7]. However, due to its initial success, several authors are now proposing this technique for idiopathic large MH as well [8]. They claim that a full-thickness retinal graft may provide a sturdier scaffold for retinal gliosis with better tissue integration and possible ectopic synaptogenesis, which may lead to better anatomical and functional outcomes [7–9].

The objectives of the current study are to assess the anatomical and functional outcomes of patients with large, refractory macular holes that have been treated with full-thickness ART, as well as to describe the structural change of macular anatomy by optical coherence tomography after 1 year of follow-up.

## Methods

Prospective, consecutive case series. The study was approved by the local internal review board. The study was conducted according to the tenets of the declaration of Helsinki and Good Clinical Practice guidelines. All sensitive data were managed according to the Health Insurance Portability and Accountability Act (HIPAA) rules. All the patients were informed about the experimental nature of the surgical technique, including a detailed description of possible complications and the main surgical alternatives. All the patients signed an informed consent form before enrollment into the study.

We included patients 18 years of age and older, with a clinical diagnosis of a persistent/refractory, open or flat-open large macular hole (minimum diameter of > 500 µm) and a history of at least 1 previous failed macular hole surgery. We excluded patients with past medical history relevant for amblyopia, diabetic retinopathy, diabetic macular edema, advanced glaucoma, age-related macular degeneration, and other macular diseases.

After enrollment, all the patients underwent a comprehensive ophthalmological examination which included assessment of the best-corrected visual acuity (BCVA), measured as the logarithm of the minimum angle of resolution (logMAR), slit lamp examination, fundus examination and optical coherence tomography (OCT, Spectralis HRA-OCT; Heidelberg Engineering, Heidelberg, Germany). The OCT images were acquired using a preset 7 Line Raster Scan of 30° × 0°, 25 frames OCT ART mean and 240-µm spacing on high resolution. If the scan was not centered, the aiming beam was manually placed in the center of the macular hole in order to ensure that the fourth of the 7 lines passed in the middle of the foveal defect. The fourth raster line was used for all measurements. From each OCT study, we assessed the integrity of the different main retinal layers, myoid and ellipsoid layers, and the Bruch-retinal pigment epithelium-choriocapillaris complex. The minimum diameter (minimal extent of the hole) and the base diameter (diameter at the level of the retinal pigment epithelium), as well as the gap defect at the level of the myoid/ellipsoid layer, were manually assessed with the measuring tool built into the software by a single observer (JCC).

All the patients had standard 23-gauge, three-port, pars plana vitrectomy (PPV) and silicone oil tamponade. All the surgeries were performed by a senior attending physician (SRJ) with the Constellation platform (Alcon Labs, Fort Worth TX, US). After the revision of the retinal periphery and removal of remaining vitreous gel, the macula was restained with brilliant blue G 0.25 mg/ml, 0.025% (Membrane Blue, Dutch Ophthalmic company, Exeter NH, US) in order to assess the ILM status and verify its absence. Additional peeling was performed in cases where remnants of ILM were noted. A full-thickness autologous retinal graft was harvested from the XII meridian, with 1 to 2 disk diameters taken from the temporal superior arcade from an area with the least amount of visible retinal vessels. The size of the graft was approximately 1.2 to 1.5 times the diameter of the MH (1000 µm approximately). Before the graft’s mechanical dissection with vertical scissors, the surgeon applied endodiathermy of the borders. With the infusion closed and under perfluorocarbon liquids, the graft was manipulated into position with ILM-forceps or diamond-dust scraper (Grieshaber, Alcon Labs, Fort Worth TX, US). The graft was placed completely into the MH (edge to edge), but the edges were not tucked under the retina. Additional encircling photocoagulation to the retinal donor site was then applied. Finally, after aspiration of the perfluorocarbon liquids and complete air-fluid exchange, the surgeon used silicon oil (5000 centistokes) as tamponade in all cases. The sclera was sutured with 7 -0 polyglactin 910 (Vicryl, Ethicon Inc, Bridgewater, NJ. USA) in all cases.

In each visit, the patients had a complete ophthalmological examination which included slit-lamp examination, intraocular pressure, and BCVA assessment and OCT examination.

Statistical analysis was done using an excel spreadsheet (Excel 2010; Microsoft Corp., Redmond, WA) with XLSTAT application v18.06 (Addinsoft, New York, NY) and the Statistical Package for Social Sciences (SPSS) software (version 20, SPSS, Inc., Chicago, IL; USA). General demographic data and OCT results are expressed in terms of means ± standard deviation. The closure rate is reported in percentage and 95% confidence intervals for a binomial distribution. BCVA progression through time was analyzed with a test for repeated dependent measurements and a Bonferroni correction, with an alpha value of 0.05 for statistical significance. Changes in the nonparametric continuous variables between baseline and the last visit was analyzed with a Mann-Whitney U test.

## Results

We included a total of 13 eyes from 13 patients (7 males, 6 females). The mean age was 67.15 ± 12.28 years-of-age. General demographic data, refraction error, and OCT baseline measurements are summarized in Table 1. The mean spherical equivalent was − 1.40 ± 3.64 and mean axial length was 25.01 ± 2.54 mm. The mean OCT measurements at baseline included a basal diameter of 1615.38 ± 689.19 µm and a minimum diameter of 964.38 ± 709.77 µm.

**Table 1:** General demographics and OCT measurements

No.	Age (years)	Gender (M/F)	Spherical equivalent (D)	Axial length (mm)	Min diameter (µm)	Base Diameter (µm)	MH status 12-months FU
1	58	M	2.25	27.09	963	1161	C
2	33	M	− 1.0	23.65	3193	1411	O
3	76	F	2.5	23.79	860	1513	C
4	69	F	− 0.5	24.43	597	956	C
5	63	F	− 1.0	23.55	847	1384	C
6	65	M	− 8.5	25.49	712	2943	O
7	81	F	− 9.75	32.55	681	1161	C
8	72	M	− 0.25	23.07	697	2856	O
9	66	M	− 1.25	23.96	583	1093	C
10	69	F	− 0.87	25.93	512	889	C
11	71	M	0.12	23.49	517	2414	C
12	82	M	0.75	24.18	1352	1665	C
13	68	F	− 0.75	23.89	1019	1554	C

No. number of cases

*M/F* Male/Female, *D* Diopters, *Min* minimum, *MH* Macular hole, *FU* Follow-up visit, *O* Open, *C* Closed

None of the patients had significant complications during surgery. Three patients experienced a transitory increase in intraocular pressure (23.08%; 95% CI 5.04–53.81%) immediately after surgery. However, all three were successfully treated with topical hypotensive drops and did not require any further intervention. The drops were suspended without incident or recurrence of high intraocular pressure after three months of follow-up. During the follow-up, one patient developed mild posterior proliferative vitreoretinopathy (grade: CP [10]), and another demonstrated a macular epiretinal membrane (7.69%; 95% CI 0.19–36.03%). The silicone oil was extracted from the vitreous cavity between the third and sixth months after the initial surgery without complications in all cases.

**Fig. 1:**
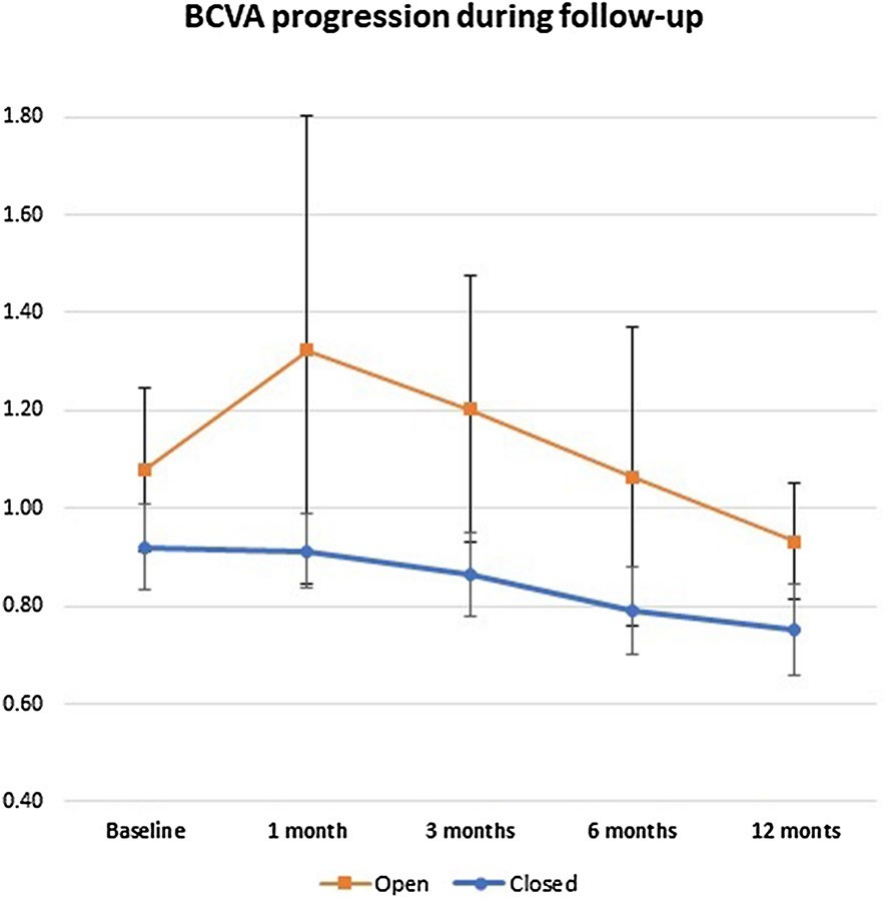
Change in visual acuity from patients with open and closed macular hole (± Standard deviation) un logMAR. *BCVA* Best-corrected visual acuity

**Fig. 2:**
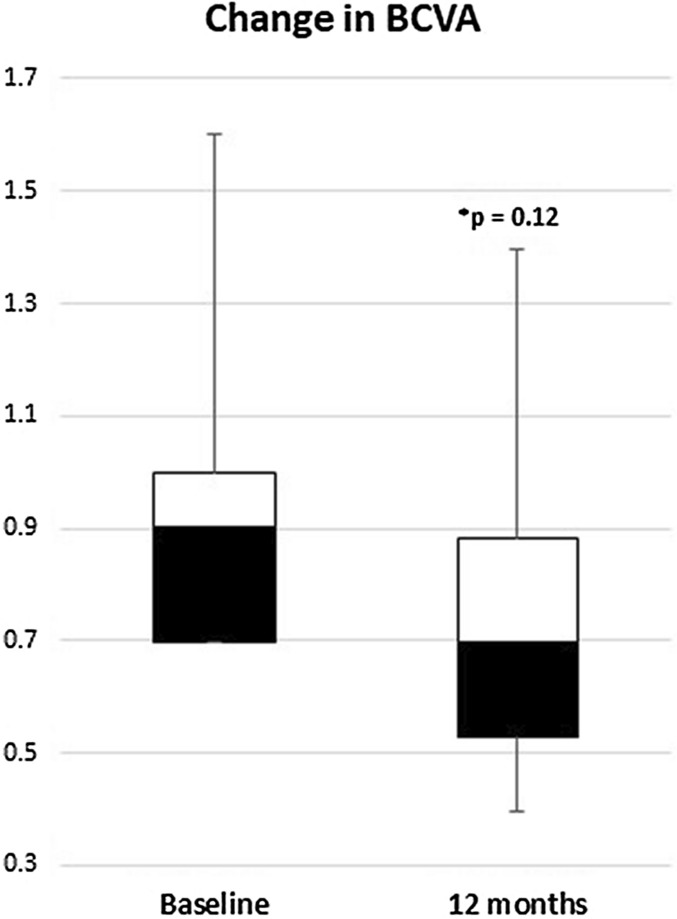
Box and whiskers plot that shows the difference in visual acuity between baseline and the 12-month follow-up measurement in logMAR. The change was not statistically significant. *BCVA* Best-corrected visual acuity

Ten out of thirteen patients had a closed MH at the 12-month follow-up visit (76.92%; 95% CI 46.19–94.96%). This group of patients had a mean BCVA at baseline of 0.92 ± 0.28 (20/166), with a median of 0.90 and a standard error of measurement (SEM) of 0.09. At the 12-month follow-up, the mean BCVA was 0.75 ± 0.29 (20/112), the median was 0.70, and the SEM was 0.09. Figure 1 describes the change in BCVA throughout time. The test for repeated measurement showed that there was a trend toward improvement, with an alpha value of 0.034. However, after the Bonferroni correction, the value lost its statistical significance. Figure 2 shows the comparison between baseline BCVA and the 12-month follow-up BCVA, and the change was not statistically significant (*p* = 0.12).

The three patients who did not achieve a closed MH at the end of the follow-up had a mean BCVA at baseline of 1.08 ± 0.17 (20/240), a median of 1.1 and SEM of 0.1. At the end of the follow-up, the mean BCVA was 0.93 ± 0.12 (20/170), with a median of 1.0 and SEM of 0.07. Although the BCVA showed a trend toward improvement throughout the follow-up, the change was not statistically significant (*p* = 0.3). In addition, patients with an open MH at the end of the follow-up tended to have worse BCVA at baseline than patients with a closed MH. However, this difference was not significant.

**Fig. 3:**
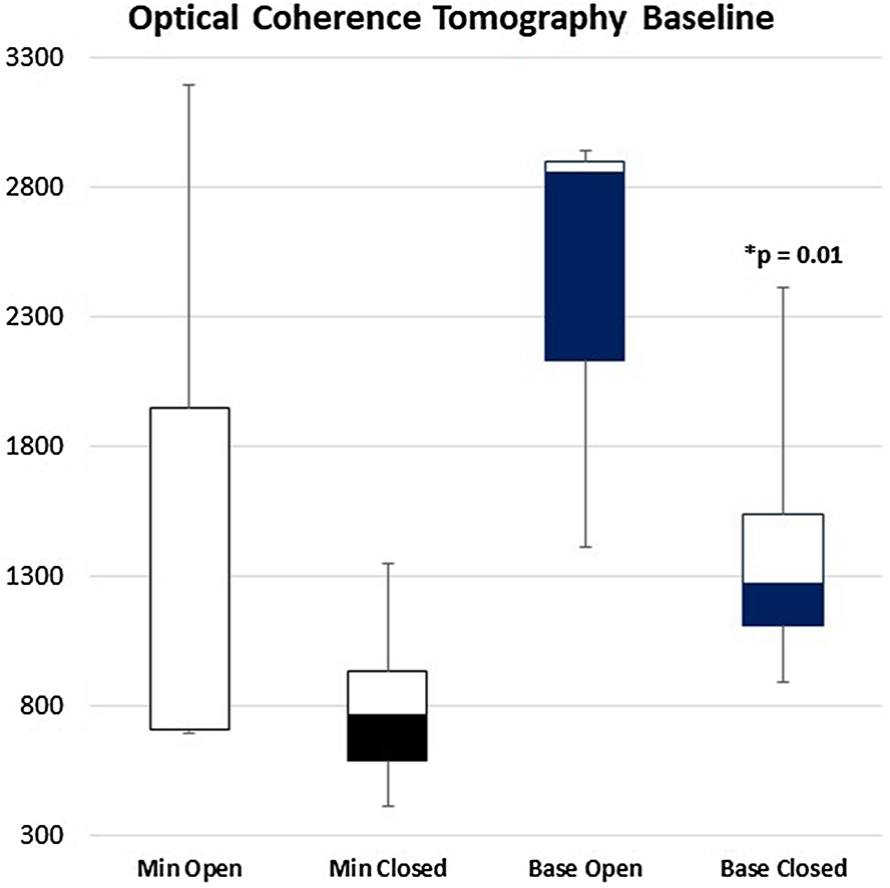
Box and whiskers plot that demonstrate the difference in OCT measurements at baseline between patients with open and closed macular holes at the end of the follow-up. *Min* minimum

There was no difference between the patients with open and closed MH at the end of the follow-up in terms of mean spherical equivalent (*p* = 02), mean axial length (*p* = 0.19), and mean minimum diameter at baseline (p *=* 0.27). However, the patients with an open MH had a significantly larger mean base diameter at baseline (2403.33 ± 860 µm vs. 1379 ± 446.9 µm; *p* = 0.01) (Fig. 3).

**Fig. 4:**
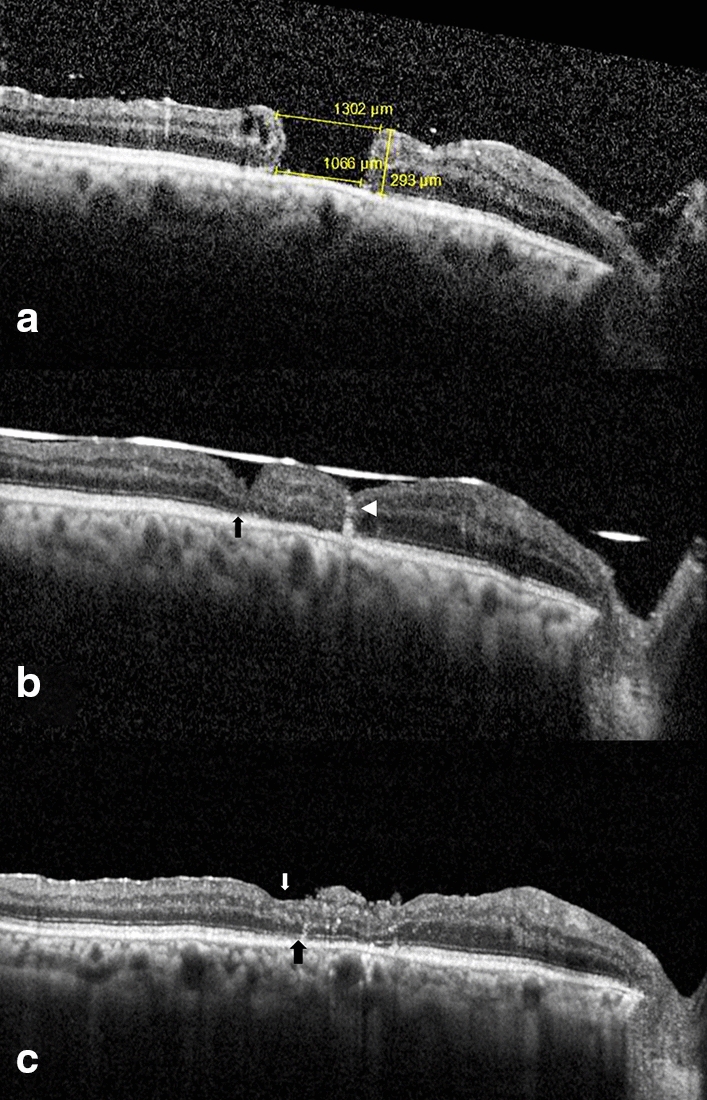
This figure shows the tissue integration and structural changes by OCT of patient No.1. **a** baseline OCT that shows a large macular hole with a mean gap on the myoid/ellipsoid layer of 1066 µm. **b** 6-months follow-up OCT of the same patient. The white arrowhead shows retinal graft integration with the edges of the macular hole. The black arrow points to a persistent defect on the myoid/ellipsoid layer. **c** 12-months follow-up OCT. The small white arrow shows an area with a pseudo-fovea formation. The black arrow shows the recovery of the myoid/ellipsoid defect and outer retinal layers normalization

Regarding OCT analysis, the patients with a closed MH at the 12-month follow-up visit had a Myoid/ellipsoid layer gap of 1277.4 ± 538.78 µm at baseline. Six patients showed full recovery of the myoid/ellipsoid layers at the end of the follow-up. The remaining four showed a reduction of 44.9% of the gap (mean: 478.5 ± 589.5 µm). All the patients with a closed MH demonstrated a full integration of the retinal graft. Nine out of ten formed a pseudofovea at the end of the follow-up with normalization of the internal retinal layers. Only one patient with an open MH at the 12-month follow-up visit had partial recovery of the myoid/ellipsoid layer with a partial integration of the retinal graft and normalization of the internal retinal layers. The other two patients showed a gap reduction of the myoid/ellipsoid layer of only 8.7% (3256 ± 1736.6 µm) (Fig. 4). None of the grafts developed macular edema during follow-up.

## Discussion

The staining and removal of the ILM with gas tamponade have become the standard of care in cases of idiopathic MHs, mainly because of its high rate of anatomical success and low risk for complications [2]. However, there are cases where the clinical presentation of the MH, along with other associated risk factors, significantly decreases the chances for surgical success. Such cases include but are not limited to, patients with abnormally large (> 400 µm), chronic (+ 6 months), or multiple MHs, MH associated with high myopia and retinal detachment, MH associated with inflammatory diseases, and MHs that failed to achieve closure, or have a flat-open configuration after one or multiple surgeries (persistent/refractory MHs) [2, 11–13].

Current strategies proposed to address such challenging cases include the use of heavy silicone oil tamponade, relaxing retinotomies and laser photocoagulation of the MH margins, temporal scleral imbrication, scleral shortening techniques, macular and suprachoroidal buckling, and the use of different types of tissues to plug the MH (blood, platelet-rich plasma, ILM, posterior lens capsule, amniotic membrane) [14–21]. Each one of these techniques has an adequate justification for its use. Nevertheless, the data supporting individual results are scarce and the lack of large randomized clinical trials prevents definitive conclusions to be drawn regarding anatomical and functional outcomes in the long term.

ILM manipulation techniques and the use of different tissue plugs are surgical techniques that have acquired increased interest recently [2]. The use of an inverted ILM flap and use of an autologous ILM free flap are two techniques proposed by Michalewska et al. and Morizane et al., respectively [19, 20, 22]. Both authors propose the use of the ILM to cover the MH and serve as a scaffold for retinal gliosis Evidence suggests that both techniques significantly improve the anatomical outcome in patients with large MH and MH associated with high myopia [19, 20, 22]. However, there is still debate regarding the long-term functional outcomes, and their implementation depends on the availability of ILM tissue. Therefore, patients with a history of failed MH surgery where the ILM has already been removed are not ideal candidates. Alternatives to this scenario include the use of posterior lens capsule or amniotic membrane to cover the MH. Both tissues provide a sturdier and more rigid graft which facilitates manipulation during surgery [18, 21, 23, 24]. Nevertheless, they are also limited by their availability (e.g., pseudophakic patients) and the obvious issues of sterility as well as manipulation from the outside to the inside of the vitreous cavity [21].

The technique proposed by Grewal and Mahmoud overcomes previous impediments encountered with the former techniques by using a full-thickness autologous retinal graft [6, 7]. The use of retinal tissue has several potential advantages over other tissues, and goes beyond serving as a scaffold and separating the retinal pigment epithelium from the vitreous cavity. Structural integration and revascularization of the retinal graft (as seen on OCT and OCT angiography studies [25]) are believed to promote centripetal migration of tissue from the MH edges and induce the restoration of the outer layers. The retinal graft may also contract and bring the MH edges closer. Finally, animal and laboratory studies have shown that retinal tissue may have the ability to rebuild functional connections between photoreceptors and bipolar or horizontal cells in a process known as ectopic synaptogenesis. In this process, new connections may allow the cones to provide input to both the rod-mediated and the cone-mediated signaling pathways due to synaptic plasticity, thus improving vision [7, 26, 27]. It has been speculated that a similar mechanism may be present during tissue remodeling of the retinal graft. However, this has yet to be proven in a clinical setting. Additional studies with microperimetry and multifocal electroretinograms will help us improve our current understanding regarding retinal tissue integration and remodeling after ART in the future.

The results of the current case series further support the use of a full-thickness retinal graft in patients with refractory MHs. The closure rate is similar to that reported by other authors, and the evidence presented by OCT analysis proves good tissue integration of the graft and restoration of the outer retinal layers. Nonetheless, conversely to previous studies including a multicenter study published by Grewal et al., we were unable to demonstrate a significant visual improvement in our patients [7]. We did observe a trend toward improvement in patients who achieved closure; however, the change lost its significance after adjusting the alpha value in order to avoid a type 1 error. The small sample size could also have affected our ability to detect small changes in visual acuity.

Interestingly, we have a patient who developed mild posterior vitreoretinopathy from the donor area and later developed an epiretinal membrane. Although this finding did not apparently affect the patient’s visual recovery, it raises concerns regarding the real safety profile of this technique. To the best of our knowledge, all the previous reports have not found any serious complications or adverse events with this technique beyond mild, transient graft edema [7–9, 25, 28, 29]. Therefore, it is possible that the true safety profile of this technique is yet to be elucidated.

The 3 patients who did not achieve a closed MH at the end of the follow-up were all males with a mean age of 56.6 ± 20.7 years. The baseline diagnosis was traumatic MH, myopic MH, and an idiopathic macular hole. Their mean base diameter was significantly larger than the mean base diameter of patients with a closed MH (2403 vs. 1379 µm). The patient with a traumatic MH developed an epiretinal membrane during the follow-up. During the first weeks after surgery, OCT images showed a retinal graft in position with signs of tissue integration. However, as the epiretinal membrane developed, it started to exert traction over the nasal border of the MH. After one month of follow-up, the retinal graft dislodged, and the MH reopened. Nevertheless, the vision remained stable and therefore the patient declined another surgery.

In the two remaining cases (myopic MH and Idiopathic MH), both the patients had persistent subretinal fluid after surgery that lasted for several weeks. The retinal grafts did not show signs of tissue integration at any point and dislodged from the recipient tissue after two months and one month of follow-up, respectively. The reasons for the graft dislodgement and its subsequent loss are unknown. However, both the patients had some degree of retinal pigment epithelium (RPE) loss. A deficient RPE pump, as well as a deficient surgical technique, may have contributed to the persistence of subretinal fluid for an excessive period of time. Finally, the lack of contact between the retinal graft photoreceptors and a healthy RPE layer may have prevented the occurrence of the necessary conditions for tissue integration.

## Conclusions

In summary, autologous full-thickness retinal transplants improve the anatomical outcome in patients with persistent/refractory MHs. The retinal autologous graft shows excellent tissue integration, recovery of the external retinal layers, and the formation of a pseudofovea in most of the successful cases. More studies are needed in order to establish the safety profile of this technique.

## Data Availability

The authors state that they have full control of all primary data (medical records) and they agree to allow *International Journal of Retina and* Vitreous, to review their data upon request.

## Abbreviations

Cs: Centistokes
MH: Macular hole
ART: Autologous full-thickness retinal transplant
HIPAA: Health Insurance Portability and Accountability Act
BCVA: Best-corrected visual acuity
logMAR: Logarithm of the minimum angle of resolution
OCT: Optical coherence tomography
ELM: Externa limiting membrane
ILM: Internal limiting membrane
PPV: Pars plana vitrectomy
SPSS: Statistical Package for Social Sciences
SEM: Standard error of measurement

## Approval code

2018R31B1.
